# Use of the HemiCAP partial hip resurfacing technique for traumatic femoral head osteochondral defects following obturator hip dislocations

**DOI:** 10.1051/sicotj/2017059

**Published:** 2018-02-21

**Authors:** Varun Arora, Pierre Navarre, Mathias Russ, Max Esser

**Affiliations:** Department of Orthopaedics, Alfred Hospital, Victoria Australia

**Keywords:** Femoral head fracture, oObturator dislocations, Femoral head osteochondral defects, Partial resurfacing, HemiCAP, Ganz dislocation

## Abstract

Fracture of the femoral head (OTA 31-C1.3) following anterior obturator dislocations are a challenging problem as the fractures are often communited, impacted and with loose osteochondral fragments, making surgical fixation difficult. This can result in residual articular defects if the fragments cannot be internally fixed and need be excised, predisposing to secondary osteoarthritis. Treatment options for these defects are limited, have variable results and with limited literature to guide us on outcomes due to the rarity of these injuries. Here, we describe the first use of the technique of partial femoral head resurfacing in two patients with such fractures and report on their long term outcomes.

## Introduction

Anterior hip dislocations comprise about 10% of traumatic hip dislocations that often occur in younger adults following high-velocity trauma mechanisms. Obturator dislocations are a variant of anterior hip dislocations that result in a shearing type injury to the weight bearing portion of the femoral head causing compression of the posterosuperior and lateral portions of the femoral head against the anteroinferior margin of the acetabulum. This causes damage to not only the articular cartilage, but also the subchondral bone producing femoral head fractures (OTA 31-C1.2) that are impacted, comminuted with possible loose osteochondral fragments, making surgical fixation challenging [1,2]. If these fractures cannot be reconstructed and are excised, the resulting impaction and osteochondral defects lead to abnormal contact forces between articulating cartilage surfaces predisposing to secondary osteoarthritis [1,2]. Treatment of focal traumatic osteochondral defects within the femoral head remains challenging and treatment recommendations are lacking in the literature.

We describe the first use of a partial resurfacing technique of the femoral head (HemiCAP; Arthrosurface, Franklin, Massachusetts, USA) for such comminuted and impacted acute femoral head fractures (OTA 31-C1.2) following obturator dislocations in two patients, with long term clinical results of five and seven years.

## Surgical technique

The obturator hip dislocation is reduced in the emergency room under sedation or in theatre. A post reduction CT scan with 3D reconstructions is obtained to assess the femoral head fracture, subchondral impaction, presence of any fragments interposed in the joint and to aid pre-operative planning.

**Figure 1:**
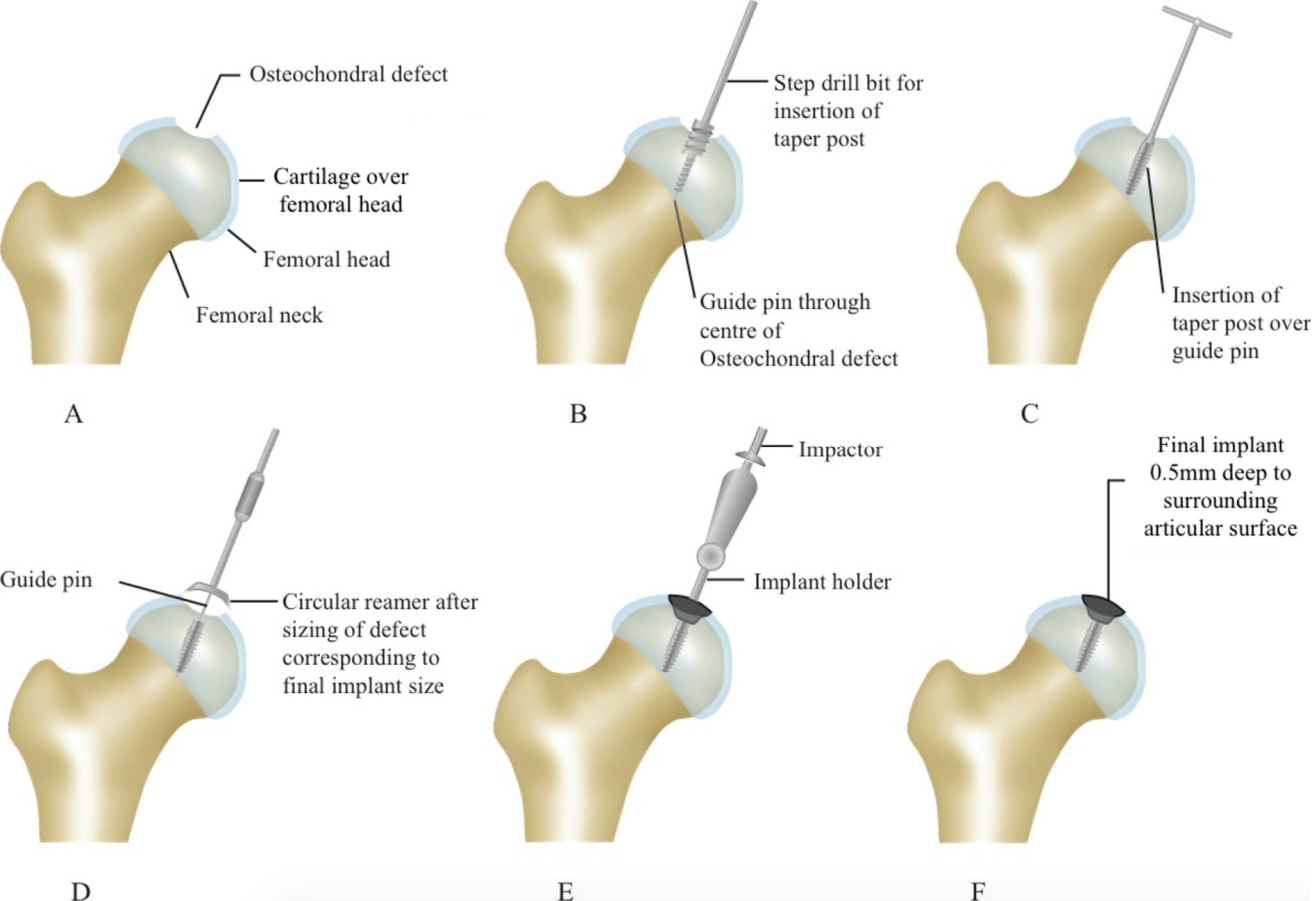
HemiCAP Hip Partial resurfacing Technique. (A) Osteochondral defect in the weight bearing portion of the femoral head. (B and C) The Centre of the defect is identified and a guidewire passed through the centre of the defect into the centre of the femoral head. A Step Drill bit is passed over the guidewire to create space to accommodate the Taper Post of the HemiCAP implant. (D) The defect is mapped and a circular reamer corresponding to the estimated size passed over the guide pin to ensure shape fit with the surrounding cartilage. (E and F) The definitive implant is placed a successful trial to ensure good congruity of the femoral head and seated flush below the surrounding articular cartilage.

The patient is positioned in a lateral decubitus position for a Ganz trochanteric flip osteotomy and a surgical hip dislocation is performed. Loose fragments are removed from the joint and the femoral head osteochondral fracture and impaction is assessed (Figure 1A). When fragments are reducible, they may be fixed with mini Acutrak (Acumed, Hillsboro, Oregon, USA), standard counter-sunk compression screws or bioabsorbable pins in anatomic position. The residual defect is measured, mapped and suitability for partial resurfacing confirmed; its centre is located and a K-wire is inserted from the centre of the lesion towards the centre of the femoral head (Figure 1B). The instrumentation allows mapping of the projected exact location of the implant and resection of the residual surface area that would allow for precise implantation. An appropriately sized HemiCAP resurfacing component is chosen and the central screw is inserted over the K-wire (Figure 1C and D). The HemiCAP is placed until it sits 0.5 mm deep to the native femoral head cartilage, without remaining proud in any location (Figure 1E and F). The hip is reduced and brought through range-of-motion under live-screening image intensifier to assess congruence and stability. The greater trochanteric flip osteotomy is stabilized with multiple partially threaded 4.0 mm cancellous screws and the wound closed in the usual manner. The patient is kept touch weight-bearing for 8–12 weeks or until consolidation of the trochanteric osteotomy.

## Cases

### Case 1

**Figure 2:**
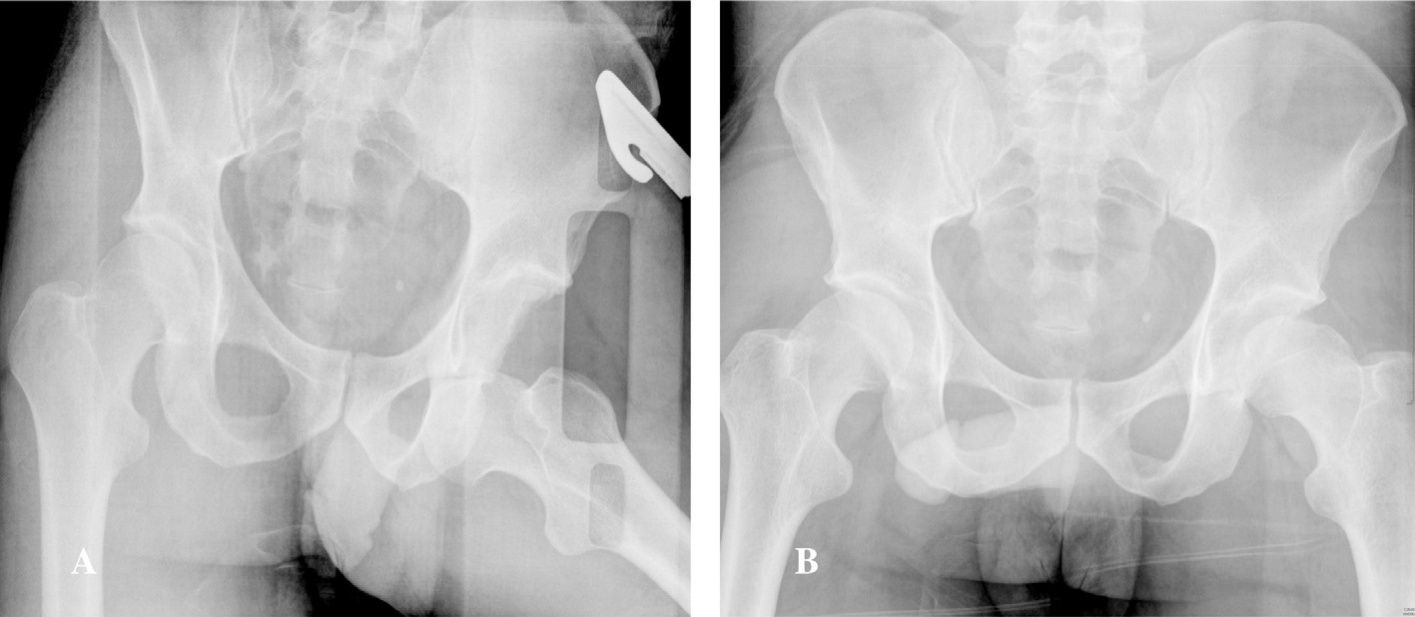
Patient 1: Anterior Obturator Dislocation (A) and Post Reduction XR (B).

**Figure 3:**
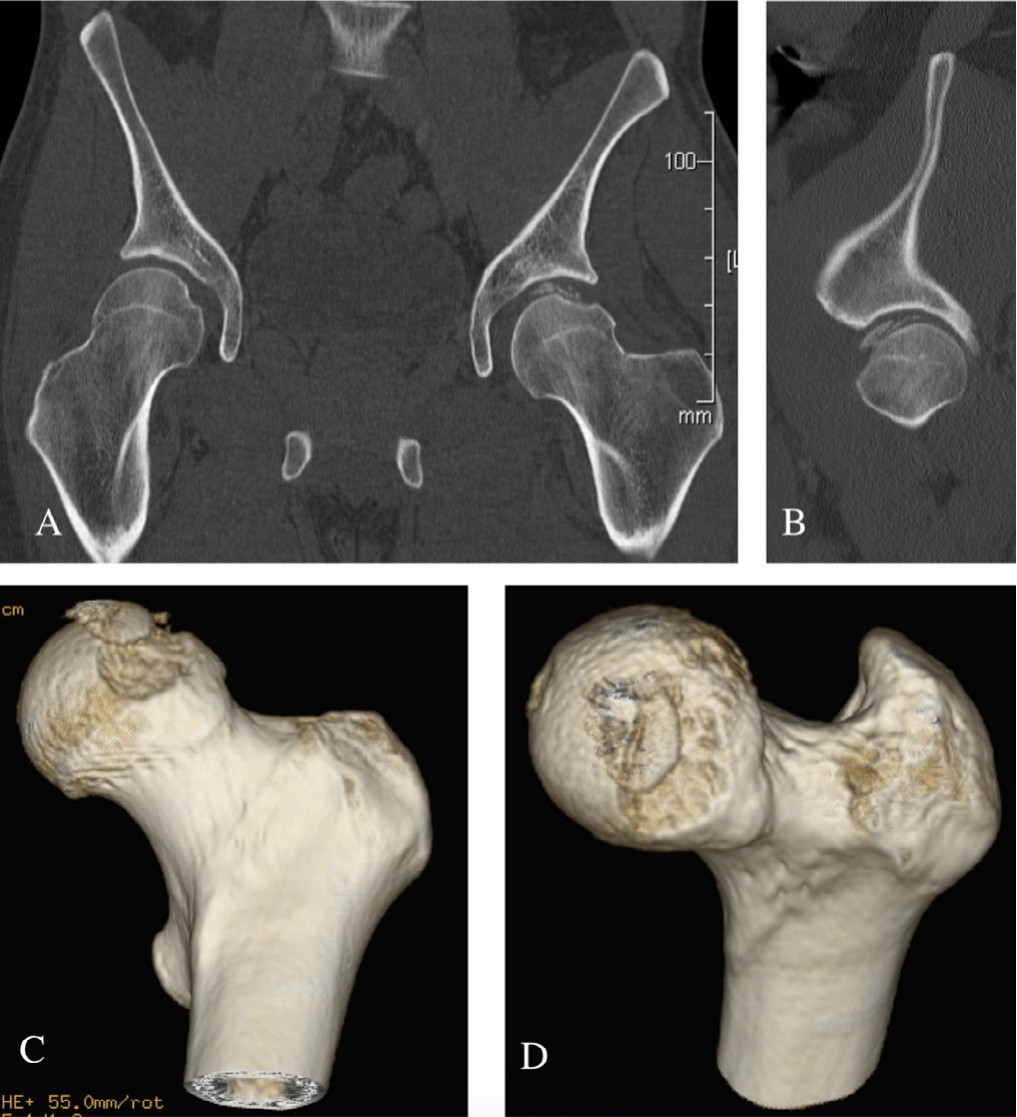
Patient 1: Post Reduction Coronal slice (A), Sagittal Slice (B) and 3D reconstruction (C,D) CT demonstrating femoral head impaction and osteochondral defect in weight bearing portion of femoral head.

**Figure 4:**
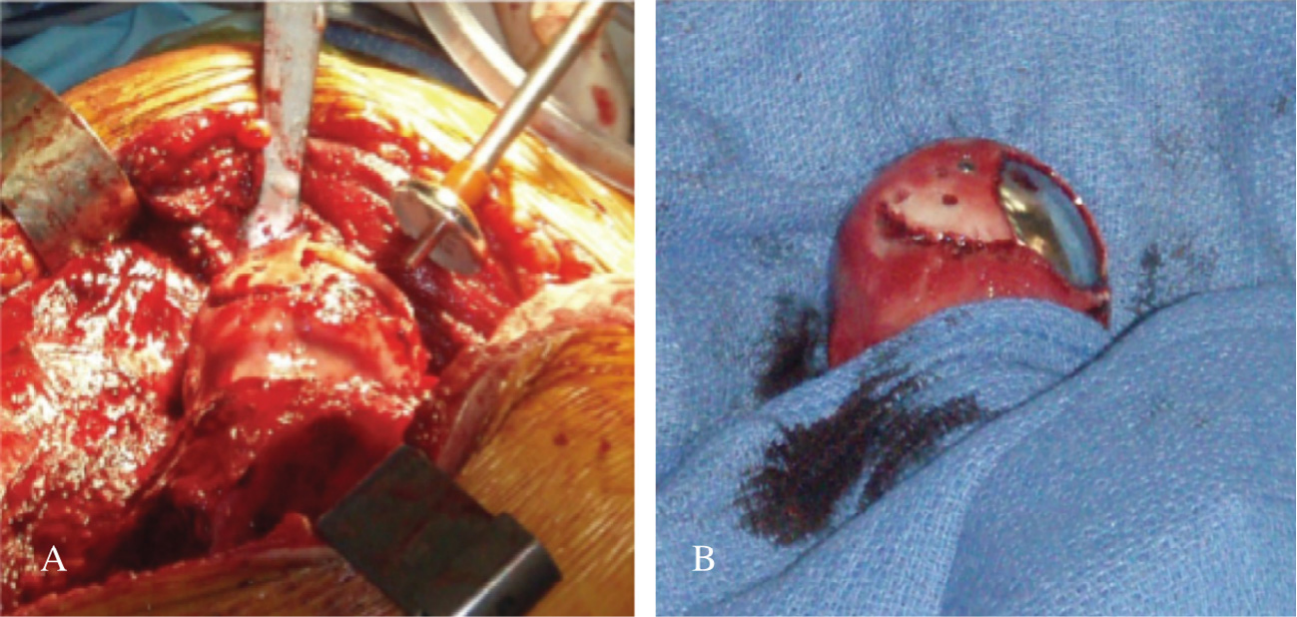
Patient 1: HemiCAP Insertion (A) and Final implant position (B).

**Figure 5:**
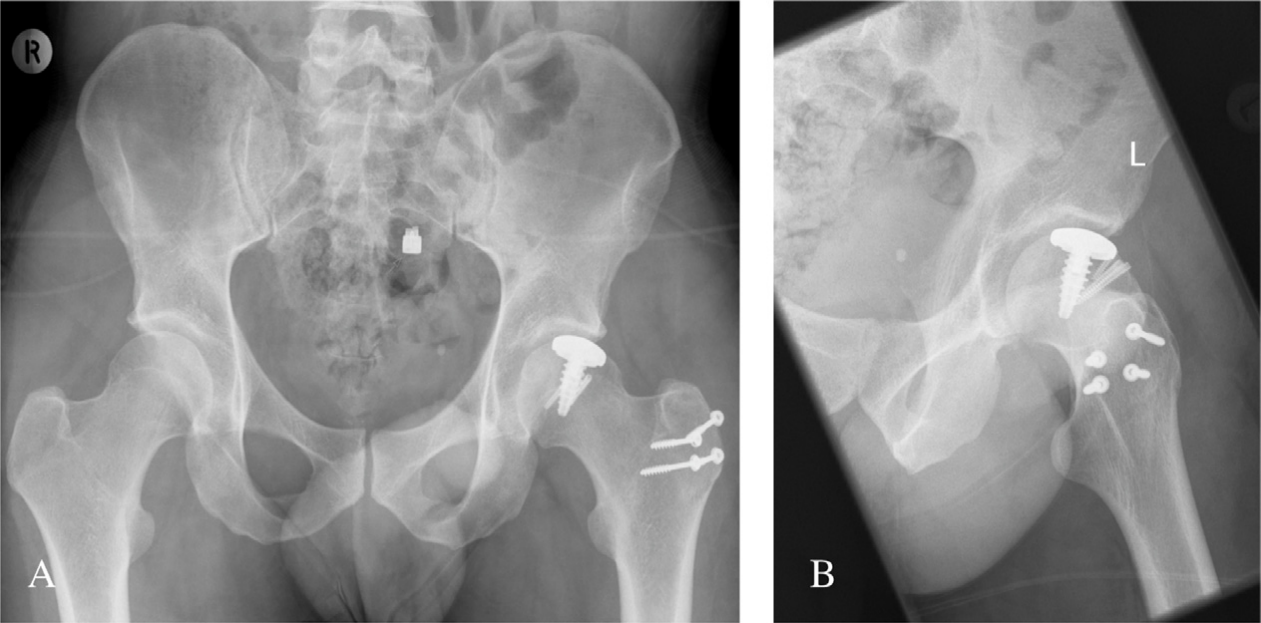
Patient 1: Final follow up X-ray at 7 years AP (A) and Lateral (B) View.

Patient 1 was a 34-years-old male involved in a high speed motor-bike accident and presented with an anterior obturator left hip dislocation (Figure 2A). He underwent a closed reduction in the emergency room within 4 h from time of injury (Figure 2B). A post-reduction CT scan demonstrated an impaction fracture of the anterosuperior aspect of the femoral head with a fragment displaced superomedially and a 25 × 25 mm impacted osteochondral defect (Figure 3). On day 6 post-admission he underwent a surgical hip dislocation using a trochanteric osteotomy. The superomedially displaced fragment was reduced anatomically and stabilized with 3×mini Acutrak screws (Acumed, Hillsboro, Oregon, USA). The adjacent residual defect within the weightbearing zone was 25 mm in diameter. The defect site was prepared and we used a 25 mm HemiCAP (HemiCAP; Arthrosurface, Franklin, Massachusetts, USA) implant to replace the defect (Figure 4). The post-operative X-rays demonstrated appropriate implant placement and a congruent joint. At 7 year follow-up he was clinically doing very well with a Harris hip score of a 100, Womac hip score of 98.4, SF-12 physical component Summary (PCS) of 56.6 and mental component summary (MCS) of 60.8. His 7-years post-operative X-rays did not demonstrate any signs of heterotopic ossification or avascular necrosis (AVN). The implant was in good position but there has been some progression of joint space narrowing with a lateral osteophyte with Tönnis grade 2 osteoarthritis (Figure 5).

### Case 2

**Figure 6:**
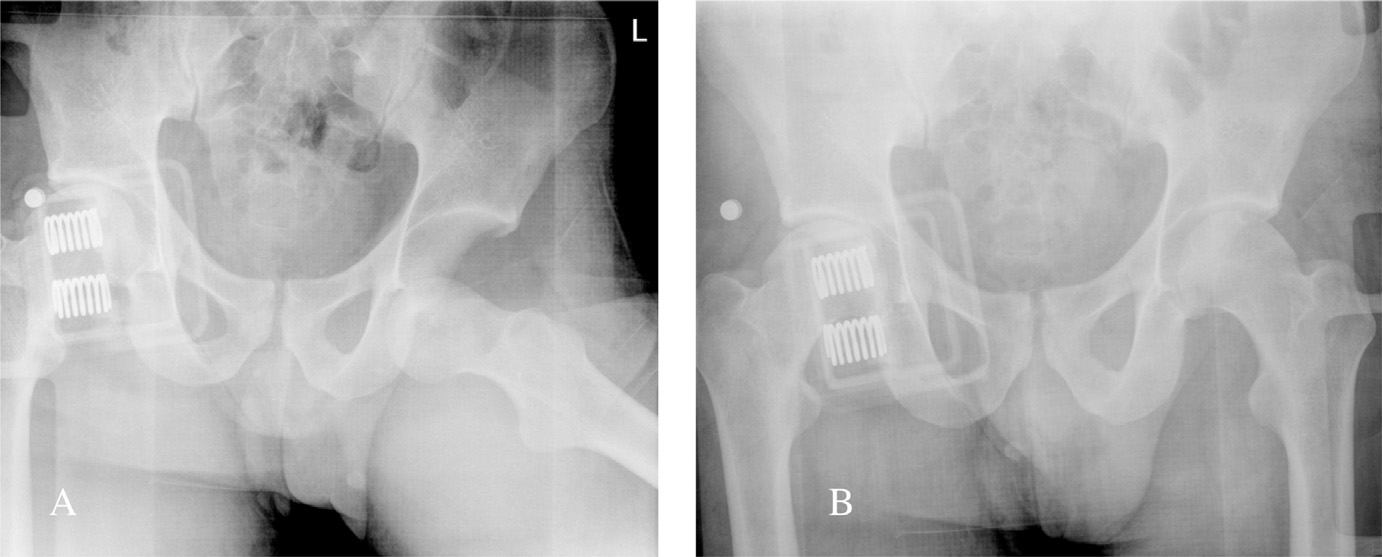
Patient 2: Anterior Obturator Dislocation (A) and Post Reduction XR (B).

**Figure 7:**
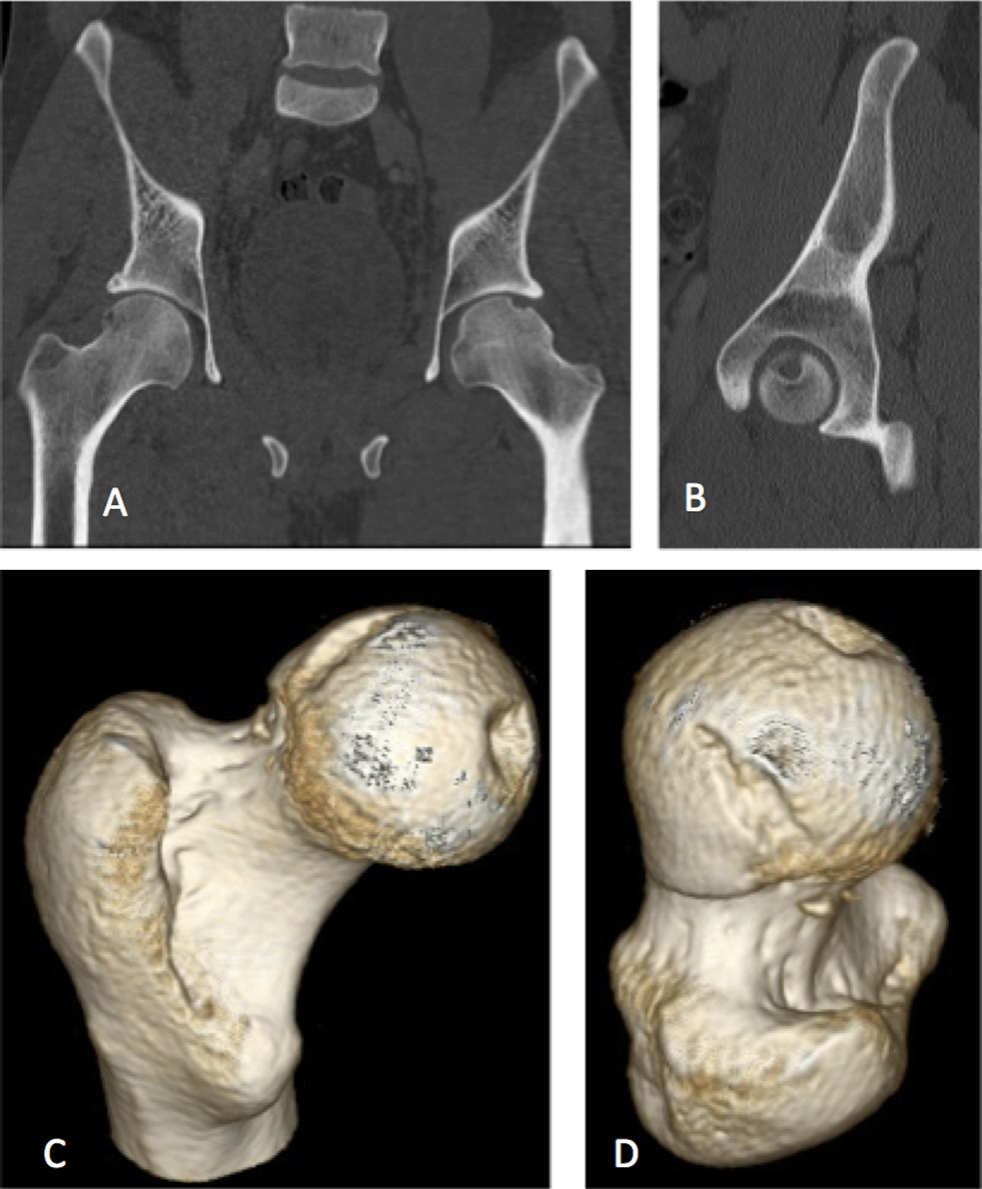
Patient 2: Post Reduction Coronal slice (A), Sagittal slice (B) and 3D reconstruction (C,D) CT demonstrating femoral head impaction and osteochondral defect in weight bearing portion of femoral head.

**Figure 8:**
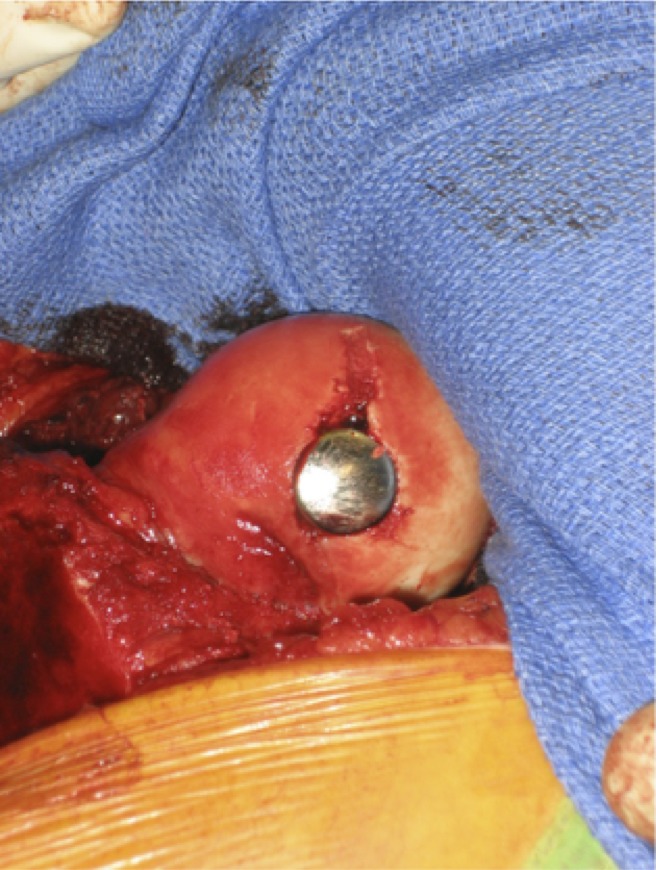
Patient 2: Final implant position.

**Figure 9:**
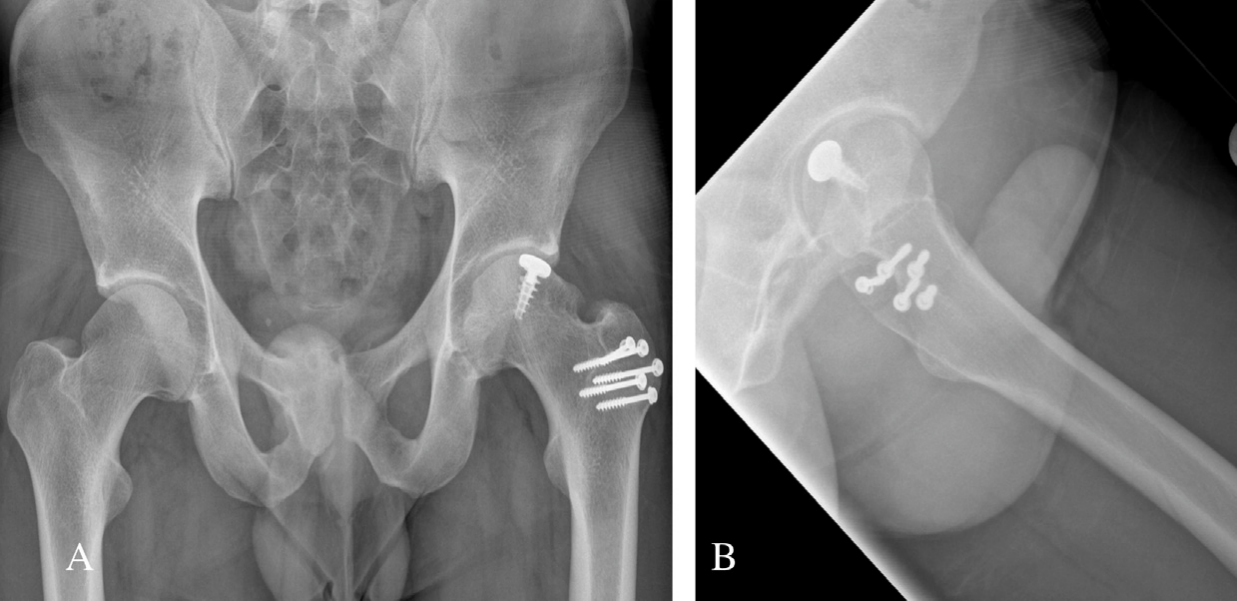
Patient 2: Final follow up X-ray at 5 years AP (A) and Lateral view (B).

Patient 2 was a 22-years old male involved in high-speed motor vehicle accident and presented with an anterior obturator left hip dislocation (Figure 6A). He underwent a closed reduction in the emergency room within 4 h from time of injury (Figure 6B). A post-reduction CT scan demonstrated loose intrarticular fragments, with a 2.5 × 2 mm osteochondral fracture and a 15 × 15 mm adjacent irregular, depressed chondral defect in the superior weight-bearing portion of the femoral head (Figure 7). On day 4 post-admission he underwent a surgical hip dislocation using a trochanteric osteotomy. The fragments were too small for fixation, therefore he underwent removal of the small osteochondral fragments, debridement of the defect site and a 15 mm HemiCAP (HemiCAP; Arthrosurface, Franklin, Massachusetts, USA) implant was used to replace the impacted osteochondral injury (Figure 8). His post-operative X-rays demonstrated appropriate implant position with a congruent joint. At 5 years follow-up he was doing well clinically with a Harris hip score of 97, Womac hip score of 98.4, SF-12 PCS of 50.3 and MCS of 59.8. His 5-years post-operative X-rays did not demonstrate any signs of heterotopic ossification or AVN. The implant was in good position with no signs of subsidence, however there is bilateral indication of femoroacetabular impingement with a lateral osteophyte and Tönnis grade 1 osteoarthritis (Figure 9).

Both patients have provided informed consent for use of their clinical, radiological and photographic material for publication purposes.

Table 1 summarizes both patients' fracture types and functional outcomes.

**Table 1:** Patient characteristics and follow-up outcome scores.

	Patient 1	Patient 2
*Patient characteristics*		
Age	34	22
Mechanism	MBA	MVA
Brumback classification	4B	4A
Approach	Ganz surgical dislocation	Ganz surgical dislocation
Defect size	25 × 25mm	15 × 15mm
HemiCAP size	25 mm	15 mm
Additional implants	3×mini-Acutrak screws	–
**Follow-up duration**	7 years	5 years
*Outcome scores*		
Harris hip score	100	97
Womac hip score	98.4	98.4
SF-12 PCS	56.6	50.3
SF-12 MCS	60.8	59.8
*Radiographic outcomes*		
OA (Tönnis Grade)	2	1
HO	Nil	Nil
AVN	Nil	Nil
Implant position	Excellent	Excellent

## Discussion

This is the first report describing the use of partial femoral head resurfacing for treatment of acute femoral head fractures.

Several authors have suggested that the prognosis for patients with femoral head fractures associated with anterior dislocations is significantly worse than those with posterior dislocations. This is because the shearing injury to the articular cartilage and subchondral bone in the weight-bearing dome can be devastating and difficult to address. It results in significant impaction, fracture comminution and presence of loose osteochondral fragments; Combined with the high contact forces across the hip joint, posttraumatic arthritis is often a rapid consequence [1,3]. Furthermore, the additional insult to the femoral head blood supply from the dislocation itself, increases the risk of AVN that further contributes to the risk of secondary osteoarthritis. magnetic resonance imaging (MRI) can identify signal changes of AVN prior to X-ray changes of subchondral sclerosis and collapse. In our series, MRIs were not done pre-operatively to look for post dislocation osteonecrosis as it is unlikely signal changes would be evident this early, but would be useful in early follow-up.

DeLee et al. [1] reported on a case series of 15 patients with obturator type of anterior hip dislocations, 13 of which had indentation or osteochondral femoral head fractures, and 10 (77%) out of the latter group developed post-traumatic osteoarthritis at average follow-up of 61 months. Furthermore, more than 50% of these patients in their series had a fair or poor outcome. They reported severe osteoarthritis and poor outcomes particularly in patients with impaction fractures deeper than 4 mm. Similarly, Erb et al. [2] reported on a series of 20 anterior hip dislocations, 7 (35%) of whom had associated femoral head fractures and described an increased incidence of post-traumatic osteoarthritis of the hip from 23% overall to 88% when additional head impaction fractures were present.

The goals of definitive management of femoral head fractures associated with anterior dislocations are to achieve an anatomically reduced femoral head with a stable hip joint and removal of any interposed bone fragments if present. Traditional surgical options include fragment excision or fixation. If a fragment is sufficiently large then fixation should be performed. Small or comminuted fragments, or fragments not within the weight-bearing portion of the head, may be excised without necessarily compromising outcome. However, If a large portion of the weight-bearing surface is involved and cannot be reconstructed because of significant comminution or impaction, options are limited. This calls for development of surgical techniques that improve clinical outcomes and prevent rapid development of post-traumatic osteoarthritis for these patients, which are often young adults.

Furthermore, it is imperative that any open approach to the hip joint in these scenarios minimizes the risk of AVN by avoiding iatrogenic injury to the branches of the medial and lateral femoral circumflex vessels that are critical for the femoral head blood supply. A Ganz surgical hip dislocation with a trochanteric flip osteotomy, like we employed in our technique, helps preserve these retinacular vessels, while still allowing adequate exposure for fracture fragment fixation or excision.

Arthroscopic microfracturing, autologous implantation, mosaicplasty by means of autologous or allograft osteochondral plugs and synthetic osteochondral substitute grafts are techniques used in reconstruction following chondral and osteochondral articular lesions. Their use in the acute traumatic situation, especially in hips, is scarce and the results mixed. Other non-chondral reconstruction techniques to deal with these weight-bearing osteochondral defects include re-directional osteotomies, prosthetic resurfacing and hip arthroplasty.

Nam et al. [4] reported on 2 cases of traumatic osteochondral defects of the femoral head treated with an osteochondral autograft taken from the knee combined with surgical dislocation of the hip. After follow-up of over 5 years, MRI studies demonstrated good autograft incorporation with maintenance of articular surface conformity. Patients complained of no pain and had a full range of motion of the hips. Similarly, Gagala et al. [5] reported on 3 cases of femoral head fracture using autologous transfer from the knee and a surgical hip dislocation, with mean Harris hip scores of 96 and mean Oxford scores of 46 at mean follow-up of 55 months. Technical challenges associated with osteochondral grafting of the hip include matching host-site geometry, regional differences in the material properties between the knee and the hip and donor site morbidity when harvested from the knee [6].

Bastien et al. [7] described the use of a local osteochondral autograft harvested from a non-weight-bearing area of the femoral head for a large traumatic osteochondral defect located within the weight bearing dome (10 × 20 mm, depth: 5 mm) following an obturator fracture-dislocation. At clinical examination 2 years postoperatively, the patient was free of pain. The function of the injured hip was comparable to that of the contralateral hip. MRI showed no signs of AVN or changes of early osteoarthritis in the rest of the hip.

If the patient is much older, then a total hip arthroplasty (THA) or hemiarthroplasty can be a successful treatment option [8,9]. THA facilitates early rehabilitation and avoids the risk of AVN and subsequent osteoarthritis, however it is not an ideal option for younger, active patients due to concerns of reduced implant survivorship [10].

In order to avoid THA, partial femoral head resurfacing techniques have been described in various other atraumatic conditions to address osteochondral defects and AVN of the femoral head. Whereas some case reports describe successful outcomes [11–13] with HemiCAP partial resurfacing arthroplasty in osteochondral lesions of the femoral head, other series are not as promising. Tzaveas et al. [14] describes a series of 12 patients treated with HemiCAP for femoral head osteochondral lesions, 5 of which required hip resurfacing arthroplasty or THA at a mean of 17 months (12–24 months) post-operatively, and only 4 patients never required further surgery with follow-up of 32.7 months. Caution was advised when using these implants for this indication. Siguier et al. [15] describe their experience in treating AVN of the femoral head with partial femoral head hemi-resurfacing using the MS prosthesis (Tornier, Saint-Ismier, France). 37 implants were used in 33 patients aged 24–59 years, 24 of which had good to excellent functional scores at a mean follow-up of 49 months. The 9 cases that failed were attributed to progression of the AVN phenomenon.

Both our patients in this present report, treated with HemiCAP partial resurfacing for obturator fracture-dislocations, had excellent results on their functional outcome scores at minimum of 5 years follow-up with only mild osteoarthritic changes on X-rays. Neither have required subsequent surgery. These results are very promising in comparison to many of the other techniques described in the literature. Although this surgical technique has been used previously to treat femoral osteochondral lesions, AVN and other uses, this is the first report to our knowledge describing its use in acute femoral head fractures.

We acknowledge that this series of 2 patients with encouraging clinical results does not represent evidence of effectiveness for this treatment, however it supports its use as an effective back-up plan in particular situations with large acute traumatic osteochondral defects recognized pre-operatively on a CT scan. In comparison to alternative reconstruction options discussed above, the operative technique for partial resurfacing is relatively straightforward without any donor-site morbidity in contrast to osteochondral autograft tehcniques.

If there is failure of treatment with implant dislodgment or post-traumatic arthritis, the subsequent treatment options would be similar to the initial salvage treatment, leading to THA. Furthermore, little bone stock loss is incurred which does not compromise eventual revision to THA.

Larger study series with long-term follow-up are required, however is limited by the rarity of these cases. From our experience with this technique, we believe that HemiCAP partial resurfacing for treatment of selected femoral head fractures in the weight-bearing portion is a useful adjunct to the known treatment options.

## Conflict of interest

The authors have no conflicts of interest to disclose.
